# Early steroids and ventilator-associated pneumonia in COVID-19-related ARDS

**DOI:** 10.1186/s13054-022-04097-8

**Published:** 2022-08-02

**Authors:** Pauline Lamouche-Wilquin, Jérôme Souchard, Morgane Pere, Matthieu Raymond, Pierre Asfar, Cédric Darreau, Florian Reizine, Baptiste Hourmant, Gwenhaël Colin, Guillaume Rieul, Pierre Kergoat, Aurélien Frérou, Julien Lorber, Johann Auchabie, Béatrice La Combe, Philippe Seguin, Pierre-Yves Egreteau, Jean Morin, Yannick Fedun, Emmanuel Canet, Jean-Baptiste Lascarrou, Agathe Delbove

**Affiliations:** 1 Service de Médecine Intensive Réanimation, Centre Hospitalier Universitaire de Nantes, 1 Place Alexis Ricordeau, 44093 Nantes Cedex 01, France; 2 Service de Réanimation Polyvalente, Centre Hospitalier Bretagne Atlantique, Vannes, France; 3 Service de Réanimation Chirurgicale, Centre Hospitalier Universitaire de Rennes, Rennes, France; 4 Plateforme de Méthodologie et Biostatistique, Centre Hospitalier Universitaire de Nantes, Nantes, France; 5 Service de Médecine Intensive Réanimation, Centre Hospitalier Universitaire d’Angers, Angers, France; 6 Service de Réanimation Polyvalente, Centre Hospitalier du Mans, Le Mans, France; 7 Service de Médecine Intensive Réanimation, Centre Hospitalier Universitaire de Rennes, Rennes, France; 8 Service de Médecine Intensive Réanimation, Centre Hospitalier Universitaire de Brest, Brest, France; 9 Service de Médecine Intensive Réanimation, Centre Hospitalier Départemental de Vendée, La Roche-sur-Yon, France; 10 Service de Réanimation Polyvalente, Centre Hospitalier de Cornouaille, Quimper, France; 11 Service de Réanimation Polyvalente, Centre Hospitalier de Saint-Malo, Saint-Malo, France; 12 Service de Médecine Intensive Réanimation, Centre Hospitalier de Saint-Nazaire, Saint-Nazaire, France; 13 Service de Réanimation Polyvalente, Centre Hospitalier de Cholet, Cholet, France; 14 Service de Réanimation Polyvalente, Centre Hospitalier Bretagne Sud, Lorient, France; 15 Service de Réanimation Polyvalente, Centre Hospitalier de Morlaix, Morlaix, France; 16 Service de Soins Intensifs de Pneumologie, Centre Hospitalier Universitaire de Nantes, Nantes, France

**Keywords:** Mechanical ventilation, SARS-CoV-19, Nosocomial pneumonia, Dexamethasone, Methylprednisolone

## Abstract

**Rationale:**

Early corticosteroid treatment is used to treat COVID-19-related acute respiratory distress syndrome (ARDS). Infection is a well-documented adverse effect of corticosteroid therapy.

**Objectives:**

To determine whether early corticosteroid therapy to treat COVID-19 ARDS was associated with ventilator-associated pneumonia (VAP).

**Methods:**

We retrospectively included adults with COVID-19-ARDS requiring invasive mechanical ventilation (MV) for ≥ 48 h at any of 15 intensive care units in 2020. We divided the patients into two groups based on whether they did or did not receive corticosteroids within 24 h. The primary outcome was VAP incidence, with death and extubation as competing events. Secondary outcomes were day 90-mortality, MV duration, other organ dysfunctions, and VAP characteristics.

**Measurements and main results:**

Of 670 patients (mean age, 65 years), 369 did and 301 did not receive early corticosteroids. The cumulative VAP incidence was higher with early corticosteroids (adjusted hazard ratio [aHR] 1.29; 95% confidence interval [95% CI] 1.05–1.58; *P* = 0.016). Antibiotic resistance of VAP bacteria was not different between the two groups (odds ratio 0.94, 95% CI 0.58–1.53; *P* = 0.81). 90-day mortality was 30.9% with and 24.3% without early corticosteroids, a nonsignificant difference after adjustment on age, SOFA score, and VAP occurrence (aHR 1.15; 95% CI 0.83–1.60; *P* = 0.411). VAP was associated with higher 90-day mortality (aHR 1.86; 95% CI 1.33–2.61; *P* = 0.0003).

**Conclusions:**

Early corticosteroid treatment was associated with VAP in patients with COVID-19-ARDS. Although VAP was associated with higher 90-day mortality, early corticosteroid treatment was not. Longitudinal randomized controlled trials of early corticosteroids in COVID-19-ARDS requiring MV are warranted.

**Supplementary Information:**

The online version contains supplementary material available at 10.1186/s13054-022-04097-8.

## Introduction

SARS-CoV-2 infection (COVID-19) is currently the leading cause of acute respiratory failure [1]. About 5% of patients develop acute respiratory distress syndrome (ARDS) requiring admission to the intensive care unit (ICU) and invasive mechanical ventilation (MV) [2]. Depending on ARDS severity, ICU mortality has ranged from 30 to 50% [3].

Ventilator-associated pneumonia (VAP) is a complication of prolonged MV whose adverse consequences include worsening hypoxemia, increases in MV duration and ICU stay length, antibiotic overuse, and higher mortality. In a prospective observational study, VAP caused 19.6% of all ICU deaths and was a contributory factor in 43.9% of deaths [4]. VAP has been reported in 28% of patients with bacterial pneumonia [5] and 43% with viral pneumonia (including cases due to H1N1) [6] requiring MV. The proportion of VAP in patients with COVID-19 ARDS was 52%, and VAP was more common in patients with COVID-19 than in those with other causes of pneumonia [7].

In severe COVID-19, a massive inflammatory response with a cytokine storm causes diffuse alveolar damage and inflammatory infiltrates [8]. Corticosteroid therapy, which suppresses inflammation, decreases 28-day mortality. Thus, in the open-label randomized RECOVERY trial, among patients receiving MV, significantly fewer died with vs. without dexamethasone (29.3% vs. 41.4%; rate ratio 0.64; 95% confidence interval [95% CI] 0.51–0.81) [9]. The double-blind CODEX trial in patients with moderate or severe COVID-19 ARDS showed mean numbers of MV-free days of 6.6 with dexamethasone and 4.0 with the placebo [10]. Dexamethasone in a dosage of 6 mg/day for 5–10 days has been recommended, with no details on the optimal time of initiation [11]. In a study of patients with H1N1 ARDS, by multivariate analysis, early corticosteroid therapy increased the risk of VAP but not the risk of death [12]. In COVID-19 ARDS, data on potential associations between early corticosteroid therapy and the risk of VAP are conflicting [13, 14].

The objective of this retrospective case–control study was to determine whether early corticosteroid therapy was associated with the incidence of VAP in patients receiving MV in the ICU for COVID-19 ARDS.

## Methods

### Study design and participants

We retrospectively identified adults who were admitted to any of 15 French ICUs in 2020 and required MV for COVID-19 ARDS. COVID-19 was confirmed by a positive reverse transcriptase-polymerase chain reaction for SARS-CoV-2 in upper and/or lower respiratory tract samples. We defined early corticosteroid therapy as the administration of systemic corticosteroids before or within 24 h after ICU admission.

### Outcomes

The main study outcome was the cumulative VAP incidence in groups with (cases) vs. without (controls) early corticosteroid therapy. Secondary outcomes were 90-day mortality, MV duration, other organ dysfunctions, and VAP characteristics.

### Data collection

At each center, the study investigator collected the data in Table 1 on standardized forms. Details are available in Additional file 1.

**Table 1:** Demographics and clinical features at ICU admission and during the ICU stay of patients with COVID-19 requiring invasive mechanical ventilation

	Total *N* = 670	Early CS^a^ *N* = 369	No early CS^a^ *N* = 301	*P* value
*Baseline characteristics*				
Age, years, mean (SD)	65.23 (10.8)	66.7 ( 10.2)	63.45 (11.3)	< 0.001
Males, *n* (%)	495 (73.9)	272 (73.7)	223 (74.1)	0.91
BMI, kg/m^2^, mean (SD)	29.7 (5.8)	30.3 ( 6.0)	28.9 (5.4)	< 0.001
Hypertension, *n* (%)	370 (55.2)	217 (58.8)	153 (50.8)	0.04
Current smoker, *n* (%)	41 (6.24)	16 ( 4.4)	25 (8.5)	0.03
Diabetes, *n* (%)	209 (31.2)	124 (33.6)	85 (28.2)	0.13
Mild-to-severe chronic renal failure, *n* (%)	65 (9.7)	43 (11.7)	22 (7.3)	0.06
Chronic pulmonary disease, *n* (%)	130 (19.4)	90 (24.4)	40 (13.3)	< 0.001
Immunosuppression, *n* (%)	107 (16.0)	75 ( 20.3)	32 (10.6)	< 0.001
Charlson Comorbidity Index, mean (SD)	3.77 (2.5)	4.34 (2.5)	3.08 (2.2)	< 0.001
*Risk factors for carriage of multidrug-resistant bacteria*				
Hospital length of stay ≥ 48 h within the past 3 months	59 (8.9)	43 (11.7)	16 (5.4)	0.0045
Antibiotics within the past 3 months	80 (12.1)	44 (12.0)	36 (12.2)	0.93
*Admission characteristics*				
Time from symptom onset to hospital admission, days, mean (SD)	8.31 (3.96)	8.26 (4.15)	8.36 (3.73)	0.74
Documented bacterial coinfection	60 (9.0)	30 (8.1)	30 (10.0)	0.41
Respiratory support on ICU day 1				< 0.001
Standard oxygen therapy	317 (48.3)	138 (37.7)	179 (61.5)	
High-flow oxygen therapy	179 (27.3)	151 (41.3)	28 (9.6)	
Noninvasive ventilation	6 (0.9)	4 (1.1)	2 (0.7)	
Invasive mechanical ventilation	155 (23.6)	73 (20.0)	82 (28.2)	
Time from ICU admission to intubation, days, mean (SD)	1.16 (2.4)	1.6 (2.9)	0.6 (1.4)	< 0.0001
Respiratory rate, breaths/min, mean (SD)	26.9 (6.3)	26.5 (6.4)	27.5 (6.1)	0.08
PaO_2_/FiO_2_, mean (SD)	143.0 (67.0)	133.1 (63.4)	155.2 (69.4)	< 0.001
CRP, mg/L, mean (SD)	174 (224)	178 (288)	170 (95)	0.70
D-Dimers, µg/L, mean (SD)	2420 (3496)	2230 (3064)	2860 (4325)	0.22
Lymphocyte count, G/L, mean (SD)	1.84 (12.32)	1.96 (15.1)	1.69 (7.21)	0.78
SOFA score, mean (SD)	5.15 (2.76)	4.9 (2.9)	5.5 (2.6)	0.0059
SAPS II, mean (SD)	38.4 (12.3)	37.8 (12.2)	39.1 (13.3)	0.22
*Treatments in the ICU*				
Days on invasive mechanical ventilation, mean (SD)	21.4 (19.1)	21.5 (20.5)	21.4 (19.1)	0.46
Neuromuscular blocking agents, *n* (%)	613 (91.7)	340 (92.1)	273 (91.3)	0.69
Prone positioning, *n* (%)	478 (71.5)	269 (72.9)	209 (69.7)	0.36
VV-ECMO, *n* (%)	45 (6.7)	23 (6.2)	22 (7.3)	0.58
Vasopressor use, *n* (%)	491 (73.5)	262 (71.2)	229 (76.3)	0.13
Renal replacement therapy, *n* (%)	113 (16.9)	56 (15.2)	57 (19.0)	0.19

ICU: intensive care unit; BMI: body mass index; CRP: C-reactive protein; SOFA: Sequential Organ Failure Assessment; SAPS II: Simplified Acute Physiology Score II; PaO_2_: arterial partial pressure of oxygen; FiO_2_: fraction of inspired oxygen; VV-ECMO: veno-venous extracorporeal membrane oxygenation

^a^Early CS was defined as the administration of systemic corticosteroid therapy before ICU admission or within 24 h after ICU admission

### Diagnosis and management of ventilator-associated pneumonia (VAP)

The criteria used to diagnose VAP are detailed in Additional file 1. Local protocols to minimize the risk of VAP included oral rather than nasal intubation whenever possible; head-of-bed elevation to 30°–45°; periodic suctioning system drainage; use of a new ventilator circuit for each patient; circuit changes only when the circuit was soiled or damaged, not routinely; replacement of the heat–moisture exchanger every 5–7 days or when soiled or malfunctioning; and daily oral hygiene [15].

### Statistical analysis

Categorical variables were described as counts (percentages) and continuous variables as mean ± SD. Normality was checked by visual inspection. For baseline characteristics, percentages were compared by applying the Chi-square test or Fisher’s exact test, as appropriate. Means were compared between groups using Student’s *t*-test or the Mann–Whitney *U* test. Two-tailed *P* values smaller than 0.05 were considered significant.

The incidence of VAP was compared between the groups with vs. without early corticosteroid therapy using the Fine and Gray competitive risk survival model, with death and extubation as the competing events [16]. To account for baseline differences in covariates, we then repeated the Fine and Gray analysis with adjustments on age, body mass index, and Charlson’s Comorbidity Index. To compare 90-day mortality between the groups with vs. without VAP, we chose the frailty model for survival data to account for the time dependency of VAP occurrence, with adjustment on age and Sequential Organ Failure Assessment (SOFA) score [17]. Antibiotic resistance of microorganisms causing VAP was rated from 0 to 3 (no resistance, MDR, XDR, PDR) and compared between groups with vs. without early corticosteroid therapy using ordinal logistic regression.

The statistical analysis was performed using SAS software (version 9.4, Cary, NC).

## Results

**Fig. 1:**
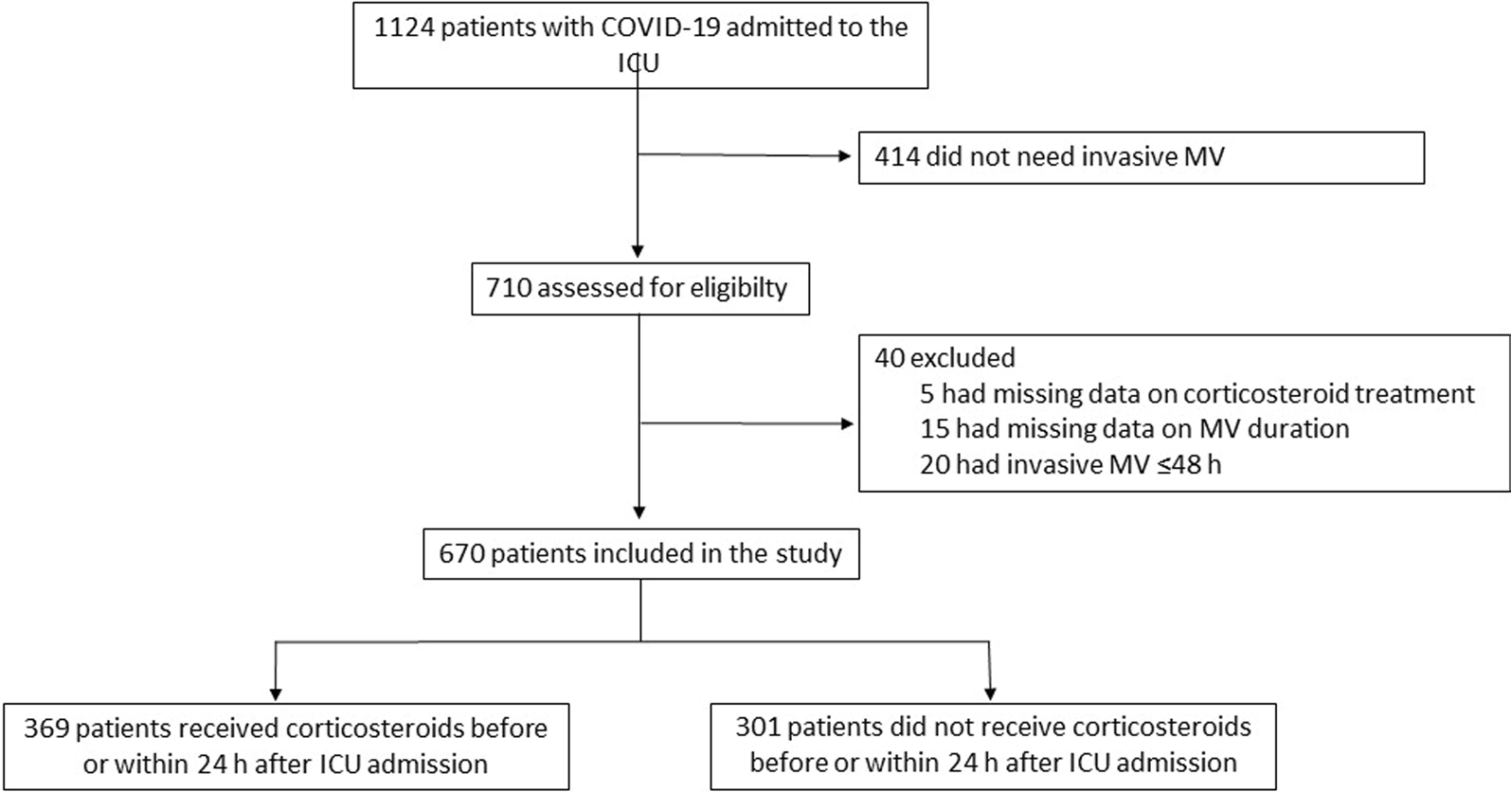
Patient flowchart

Between February 1 and December 31, 2020, 1124 patients were admitted to one of the 15 participating ICUs for COVID-19 ARDS, including 670 patients who required MV for longer than 48 h. Of these 670 patients, 369 did and 301 did not receive early corticosteroid therapy (Fig. 1).

### Demographics and characteristics

Table 1 reports the main patient features. The median time from COVID-19 symptom onset to ICU admission was 8 ± 4 days. Overall, the patients had severe hypoxemia, with a mean PaO_2_/FiO_2_ of 143 ± 67 mmHg at ICU admission.

### Details of corticosteroid treatment

In the early corticosteroid group, dexamethasone was the most frequently used form (336/369, 91%), the mean prednisone-equivalent dosage was 60 ± 48 mg, and the median corticosteroid treatment duration was 10 ± 4 days. Treatment was initiated 0 (IQR: [− 1;0]) days after ICU admission. Of the 301 other patients, 53 (17.6%) received delayed corticosteroid therapy, with initiation at a mean of 13 ± 9 days after ICU admission; methylprednisolone was the most commonly used corticosteroid (19/53, 36%), the mean prednisone-equivalent dose was 171 ± 207 mg, and the mean duration was 9 ± 11 days. Adjuvant immunomodulatory drugs such as IL-6 antagonists were used in 10 (3%) patients with vs. none of the patients without early corticosteroid therapy.

### Ventilator-associated pneumonia (VAP) (Table 2)

**Table 2:** Characteristics of ventilator-associated pneumonia (VAP in the groups with vs. without early corticosteroid therapy

	Total *N* = 670	Early CS^a^ *N* = 369	No early CS^a^ *N* = 301	*P* value
At least one VAP episode, *n* (%)	349 (52.1)	202 (54.7)	147 (48.8)	–
Days from intubation to 1st VAP episode onset, mean (SD)	9.9 (7.7)	9.9 (9.0)	10.0 (5.5)	0.11
Active empirical antibiotic treatment, *n* (%)	259 (78.3)	156 (80.8)	103 (74.6)	0.1370
Abscess, *n* (%)	13 (2.0)	8 (2.2)	5 (1.7)	0.6612
Pleural effusion, *n* (%)	7 (1.05)	5 (1.4)	2 (0.7)	0.4710
Bacteremia during VAP, *n* (%)	32 (4.8)	22 (6.1)	10 (3.3)	0.1891
2nd VAP episode, *n* (% patients with 1st VAP episode)	117 (33.5)	67 (33.2)	50 (34.0)	0.6803
Bacterial antibiotic resistance profile for 1st VAP episode (*n* = 199 and *n* = 147 with vs. without early CS)				0.0751
Normal, *n* (%)	240 (72.5)	139 (72.8)	101 (72.1)	
MDR, *n* (%)	74 (22.4)	44 (23.0)	30 (21.4)	
XDR, *n* (%)	17 (5.1)	8 (4.2)	9 (6.4)	
PDR, *n* (%)	0 (0.0)	0 (0.0)	0 (0.0)	
Missing data, *n* (%)	15 (4.3)	8 (4.2)	7 (4.8)	

CS: corticosteroid therapy; VAP: ventilator-associated pneumonia; ICU: intensive care unit; MDR: multidrug-resistant; XDR: extensively drug-resistant; PDR: pandrug-resistant

^a^Early CS was defined as the administration of systemic corticosteroid therapy before ICU admission or within 24 h after ICU admission

Of 660 respiratory tract samples, 466 produced positive bacterial cultures. The mean number of samples per patient was 2.3 ± 2.5 in the group with vs. 1.9 ± 1.9 in the group without early corticosteroids (*P* = 0.017). Of the 670 patients, 349 (52%) experienced at least one VAP episode. Mean time from intubation to the first episode was 9.9 ± 7.7 days. The proportions of patients with a first VAP episode who experienced a second episode were similar in the two groups. The bacterial antibiotic resistance profiles for the first VAP episode were also similar, with an odds ratio of 0.942 (95% confidence interval [95% CI] 0.58–1.52; *P* = 0.80) (Additional file 1).

Bloodstream infection (BSI) diagnosed during VAP was not significantly more common in the early corticosteroid group, and neither were significant differences observed for the occurrence of abscess or pleural effusion (Table 2).

**Fig. 2:**
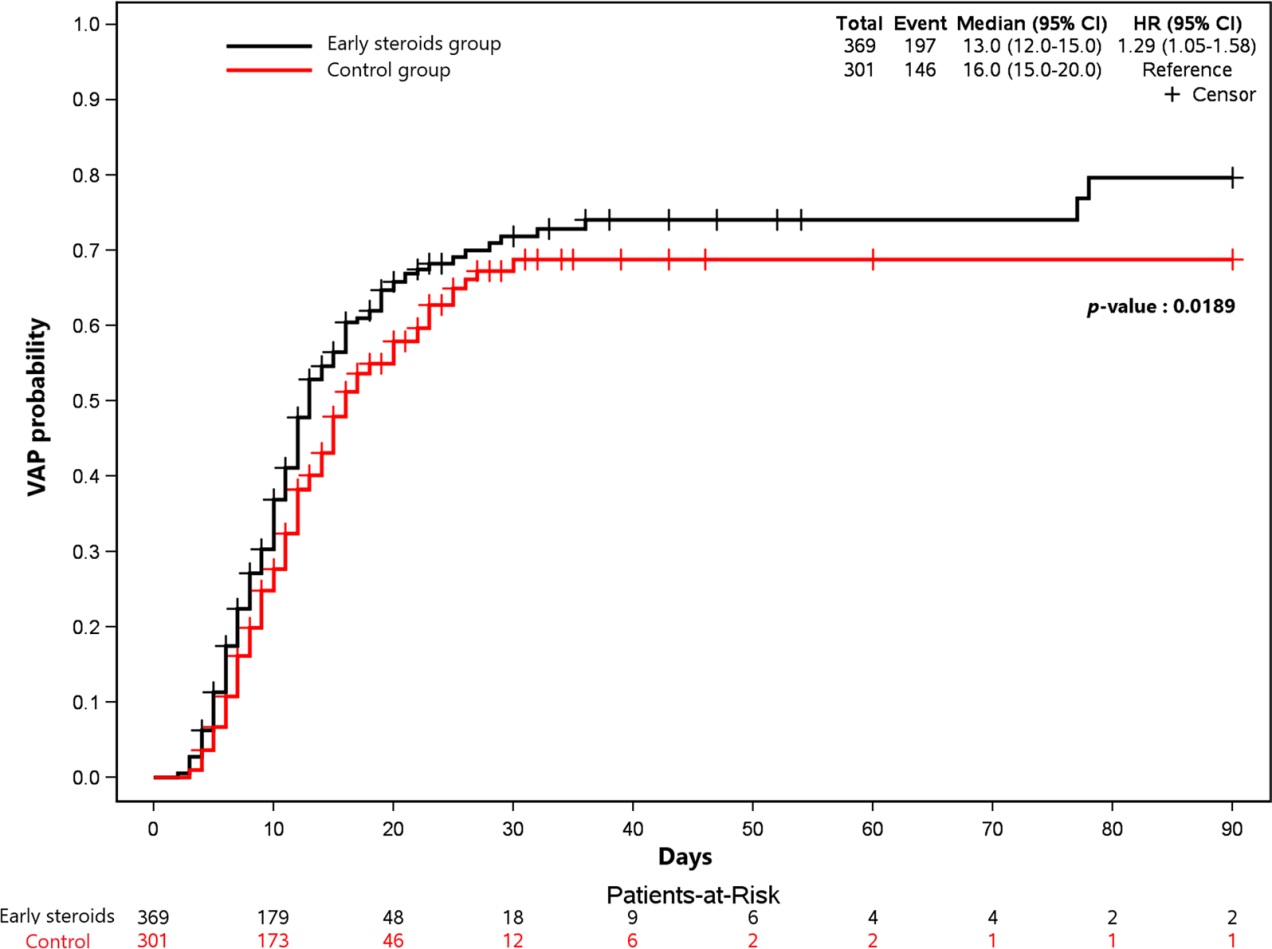
VAP probability according to time

The Fine and Gray competitive risk model with death and extubation as the competing events showed that early corticosteroid treatment was significantly associated with VAP (hazard ratio [HR] 1.29; 95% CI 1.05–1.58; *P* = 0.016) (Fig. 2). The results were similar after adjustment on age, body mass index, and Charlson’s Comorbidity Index (HR 1.28; 95% CI 1.03–1.58; *P* = 0.026).

### Bloodstream infection (BSI)

BSI diagnosed in the absence of VAP was significantly more common in the group with vs. without early corticosteroid therapy (18.7% vs. 12.7%, *P* = 0.03) (Additional file 1). The antibiotic resistance profiles of bacteria from blood cultures were similar in the two groups, as detailed in Additional file 1.

### Mortality

**Fig. 3:**
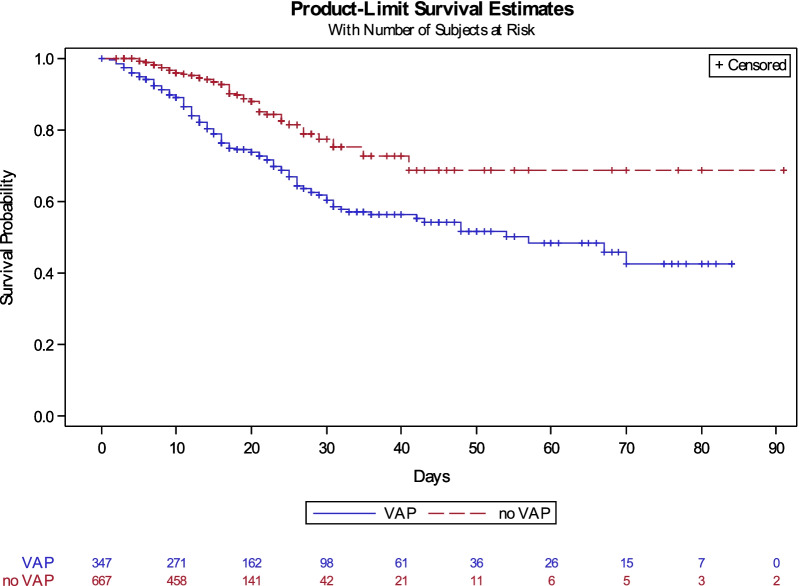
Cumulative incidence of death according to the occurrence of VAP with extubation as a competing event

Overall, 187 (27.9%) patients died in the ICU or hospital before day 90 (Table 3, Additional file 1). The frailty model with adjustment on age and SOFA score indicated that VAP was associated with higher 90-day mortality (HR 1.86; 95% CI 1.33–2.61; *P* = 0.0003). Each 1-year increase in age and each 1-point increase in the SOFA score was significantly associated with higher 90-day mortality (HR 1.05; 95% CI 1.03–1.07; *P* < 0.0001; and HR 1.15; 95% CI 1.09–1.22; *P* < 0.0001; respectively (Fig. 3).

**Table 3:** Outcomes in the groups with and without early corticosteroid therapy

	Total *N* = 670	Early CS^a^ *N* = 369	No early CS^a^ *N* = 301	*P* value
*Vital status on day 28*				
Dead, *n* (%)	129 (19.3)	76 (20.6)	53 (17.6)	0.68
Alive, out of the ICU, *n* (%)	329 (49.1)	173 (46.8)	156 (51.8)	
Alive, still in the ICU, *n* (%)	212 (31.6)	120 (32.5)	92 (30.6)	
*Vital status on day 90*				
Dead, *n* (%)	187 (27.9)	114 (30.9)	73 (24.3)	0.12
Alive, out of the ICU, *n* (%)	469 (70.0)	246 (66.7)	223 (74.1)	
Alive, still in the ICU, *n* (%)	14 (2.1)	9 (2.4)	5 (1.7)	

Early CS was defined as the administration of systemic corticosteroid therapy before ICU admission or within 24 h after ICU admission

CS: corticosteroid therapy; ICU: intensive care unit

## Discussion

In this large retrospective cohort, both VAP and BSI were more common in patients with vs. without early corticosteroid treatment given to treat severe COVID-19 ARDS. The risk of death by day 90 was higher in patients with VAP and was not lower in those given early corticosteroid treatment. Bacterial resistance profiles did not differ between the groups with vs. without early corticosteroid therapy.

The incidence of VAP in patients with COVID-19 has varied from 40 to 60% [13, 14, 18]. This range is higher than in patients with ARDS not related to COVID-19 [19]. Possible explanations to this high VAP incidence may include insufficient human and material resources to deal with surges of patients requiring MV for COVID-19 [20]. However, during the first COVID-19 wave in France, in an area where ICU bed numbers were sufficient and nurse–patient ratios unaltered, VAP developed at least once in 50% of patients with COVID-19-ARDS [13]. Moreover, the lymphopenia and cytokine storm caused by the SARS-CoV-2 virus may induce lung–parenchyma lesions conducive to bacterial growth [21]. The reported association linking VAP to higher 28-day mortality in COVID-19-ARDS but not in other causes of ARDS supports a role for specific SARS-CoV-2-generated lung lesions in inducing greater VAP severity [22]. We assessed infections and mortality until day 90, whereas other studies usually stopped data collection on day 28 [23, 24], notably a randomized clinical trial on the effect of corticosteroid therapy [10].

After publication of the RECOVERY trial showing lower 28-day mortality in COVID-19 patients receiving respiratory support at randomization and given dexamethasone [9], systemic corticosteroids became a major component of the management of patients with severe COVID-19 ARDS. In the CODeX randomized controlled trial in patients with moderate-to-severe COVID-19, dexamethasone increased the number of days alive without ventilation compared to standard care alone, and the proportions of patients with VAP were similar in the two groups [10]. However, data were collected only until day 28.

Several previous studies did not find a higher risk of VAP in patients given early corticosteroid therapy for COVID-19. In a single-center retrospective study of 135 patients admitted to the ICU for COVID-19, the groups with vs. without corticosteroid therapy did not differ significantly regarding the frequency of respiratory, urinary, or bloodstream infections [18]. Similarly, a retrospective comparison of the first and second COVID-19 waves in France found that, in patients who required MV for at least 48 h, dexamethasone therapy was not independently associated with VAP or BSI [25]. However, the time from intubation to VAP was shorter in the corticosteroid group, and the sample (*n* = 151) was five times smaller than ours. In a retrospective analysis of data collected prospectively in 70 ICUs, corticosteroid therapy within one day after ICU admission was not associated with VAP by multivariable analysis (adjusted odds ratio [aOR] 1.05; 95% CI 0.83–1.34] [23]. Other studies did find that corticosteroids were associated with VAP. A single-center retrospective study that included first-wave patients in Spain demonstrated a significant independent association of corticosteroids with VAP (aOR 3.23; 95% CI 1.78–5.97) [14]. In a prospective international study of 3777 patients, propensity-score analysis demonstrated that early dexamethasone was significantly associated with VAP (17.1% vs. 13.2%; *P* = 0.014) [26]. The best-designed studies with the largest sample sizes would thus seem to support an association linking corticosteroid therapy to VAP. The apparent discrepancies across studies may be related to differences in the timing and dosage of corticosteroid therapy and in the mix of COVID-19 phenotypes as defined, for instance, by age and inflammation severity. A study of a prospective database showed that early corticosteroid therapy was associated with lower mortality in patients aged 60 years or older but with higher mortality in those who were younger than 60 years and had laboratory evidence of inflammation [27]. A cluster analysis of prospective data from 63 ICUs in Spain identified three phenotypes that differed regarding variables including age, inflammation severity, and ICU mortality [28]. Another cohort study, done in France and Belgium, also identified three clinical phenotypes associated with different outcomes [29]. Finally, in a prospective cohort investigated using latent class analysis of respiratory parameters, no phenotypes were differentiated at baseline, whereas two phenotypes were identified when data over the first 4 days of MV were considered [30]. Further work to identify phenotypes and the optimal treatments for each is needed [31].

In our study, ICU mortality was not significantly different with vs. without corticosteroid therapy. In a retrospective multicenter study of prospectively collected data in patients similar to ours, mortality was lower with early corticosteroid therapy but higher with corticosteroids started after 17 ICU days [23]. Similarly, corticosteroid therapy started at least 13 days after symptom onset (a median of 11 [8–16] days after intubation) was not associated with lower mortality in a multicenter retrospective study [32]. However, both studies were done early in the pandemic, at a time when corticosteroids were used in higher dosages in compliance with recommendations for the treatment of ARDS [33].

The main limitation of our study is the retrospective design. However, we used a strong statistical strategy to compare patients with vs. without corticosteroid therapy. The diagnosis of VAP, known to be challenging given the nonspecific manifestations, was not validated by an adjudication committee. Nonetheless, European Centre for Disease Prevention and Control criteria for VAP were applied [34]. Although these criteria are not fully sensitive or specific, no better diagnostic method exists to date [35]. Importantly, corticosteroid treatment was not allocated at random, and significant baseline differences existed between the two groups. Thus, the group given early corticosteroid therapy was characterized by greater disease severity, a higher frequency of BSI, and a higher proportion of patients managed with high-flow nasal oxygen and delayed MV. These differences suggest that corticosteroids were given to the sickest patients, resulting in selection and misclassification bias. Finally, our study took place before the circulation of variants of the Wuhan-Hu-1 virus. The response to corticosteroids and other treatments may have changed with the emergence of variants.

## Conclusion

VAP, as well as BSI, was more common among patients given early corticosteroid therapy to treat COVID-19 ARDS requiring MV. 90-day mortality was not associated with early corticosteroid therapy but was higher in patients with VAP. Further studies should seek to identify patient subgroups likely to benefit from early corticosteroid therapy in the current era of increasing IL-6 antagonist use and emerging variants. Also, the optimal time of corticosteroid initiation should be determined.

## Supplementary Information

**Additional file 1:** Supplementary methods, eFigure 1, eTable 1 and eFigure 2.

## Data Availability

The study data will be available from the corresponding author upon reasonable request.

## References

[1] COVID-19 situation update worldwide, as of week 2, updated 20 January 2022. Eur Cent Dis Prev Control [cited 2022 Jan 21]. https://www.ecdc.europa.eu/en/geographical-distribution-2019-ncov-cases.

[2] Grasselli G, Zangrillo A, Zanella A, Antonelli M, Cabrini L, Castelli A (2020). Baseline characteristics and outcomes of 1591 patients infected with SARS-CoV-2 admitted to ICUs of the Lombardy Region, Italy. JAMA.

[3] COVID-ICU Group on behalf of the REVA Network and the COVID-ICU Investigators (2020). Clinical characteristics and day-90 outcomes of 4244 critically ill adults with COVID-19: a prospective cohort study. Intensive Care Med.

[4] Koulenti D, Tsigou E, Rello J (2017). Nosocomial pneumonia in 27 ICUs in Europe: perspectives from the EU-VAP/CAP study. Eur J Clin Microbiol Infect Dis Off Publ Eur Soc Clin Microbiol.

[5] Forel JM, Voillet F, Pulina D, Gacouin A, Perrin G, Barrau K (2012). Ventilator-associated pneumonia and ICU mortality in severe ARDS patients ventilated according to a lung-protective strategy. Crit Care.

[6] Razazi K, Arrestier R, Haudebourg AF, Benelli B, Carteaux G, Decousser J (2020). Risks of ventilator-associated pneumonia and invasive pulmonary aspergillosis in patients with viral acute respiratory distress syndrome related or not to Coronavirus 19 disease. Crit Care.

[7] Llitjos J-F, Bredin S, Lascarrou J-B, Soumagne T, Cojocaru M, Leclerc M (2021). Increased susceptibility to intensive care unit-acquired pneumonia in severe COVID-19 patients: a multicentre retrospective cohort study. Ann Intensive Care.

[8] Soy M, Keser G, Atagündüz P, Tabak F, Atagündüz I, Kayhan S (2020). Cytokine storm in COVID-19: pathogenesis and overview of anti-inflammatory agents used in treatment. Clin Rheumatol.

[9] RECOVERY Collaborative Group, Horby P, Lim WS, Emberson JR, Mafham M, Bell JL, et al. Dexamethasone in hospitalized patients with Covid-19—preliminary report. N Engl J Med. 2020. 10.1056/NEJMoa2021436 PMC7383595 32678530

[10] Tomazini BM, Maia IS, Cavalcanti AB, Berwanger O, Rosa RG, Veiga VC (2020). Effect of dexamethasone on days alive and ventilator-free in patients with moderate or severe acute respiratory distress syndrome and COVID-19: the CoDEX randomized clinical trial. JAMA.

[11] Nasa P, Azoulay E, Khanna AK, Jain R, Gupta S, Javeri Y (2021). Expert consensus statements for the management of COVID-19-related acute respiratory failure using a Delphi method. Crit Care Lond Engl.

[12] Martin-Loeches I, Lisboa T, Rhodes A, Moreno RP, Silva E, Sprung C (2011). Use of early corticosteroid therapy on ICU admission in patients affected by severe pandemic (H1N1)v influenza A infection. Intensive Care Med.

[13] Blonz G, Kouatchet A, Chudeau N, Pontis E, Lorber J, Lemeur A (2021). Epidemiology and microbiology of ventilator-associated pneumonia in COVID-19 patients: a multicenter retrospective study in 188 patients in an un-inundated French region. Crit Care Lond Engl.

[14] Martínez-Martínez M, Plata-Menchaca EP, Nuvials FX, Roca O, Ferrer R (2021). Risk factors and outcomes of ventilator-associated pneumonia in COVID-19 patients: a propensity score matched analysis. Crit Care.

[15] Hua F, Xie H, Worthington HV, Furness S, Zhang Q, Li C (2016). Oral hygiene care for critically ill patients to prevent ventilator-associated pneumonia. Cochrane Database Syst Rev.

[16] Gooley TA, Leisenring W, Crowley J, Storer BE (1999). Estimation of failure probabilities in the presence of competing risks: new representations of old estimators. Stat Med.

[17] 9781420073881: Frailty models in survival analysis (Chapman & Hall/CRC biostatistics series)—AbeBooks—Wienke, Andreas: 1420073885 [cited 2022 Mar 21]. https://www.abebooks.com/9781420073881/Frailty-Models-Survival-Analysis-Chapman-1420073885/plp.

[18] Ritter LA, Britton N, Heil EL, Teeter WA, Murthi SB, Chow JH (2021). The impact of corticosteroids on secondary infection and mortality in critically ill COVID-19 patients. J Intensive Care Med.

[19] Rouzé A, Martin-Loeches I, Povoa P, Makris D, Artigas A, Bouchereau M (2021). Relationship between SARS-CoV-2 infection and the incidence of ventilator-associated lower respiratory tract infections: a European multicenter cohort study. Intensive Care Med.

[20] Hoogendoorn ME, Brinkman S, Bosman RJ, Haringman J, de Keizer NF, Spijkstra JJ (2021). The impact of COVID-19 on nursing workload and planning of nursing staff on the Intensive Care: a prospective descriptive multicenter study. Int J Nurs Stud.

[21] Boumaza A, Gay L, Mezouar S, Bestion E, Diallo AB, Michel M (2021). Monocytes and macrophages, targets of severe acute respiratory syndrome coronavirus 2: the clue for coronavirus disease 2019 immunoparalysis. J Infect Dis.

[22] Nseir S, Martin-Loeches I, Povoa P, Metzelard M, Du Cheyron D, Lambiotte F (2021). Relationship between ventilator-associated pneumonia and mortality in COVID-19 patients: a planned ancillary analysis of the coVAPid cohort. Crit Care.

[23] Moreno G, Carbonell R, Martin-Loeches I, Solé-Violán J, Correig I Fraga E, Gómez J (2021). Corticosteroid treatment and mortality in mechanically ventilated COVID-19-associated acute respiratory distress syndrome (ARDS) patients: a multicentre cohort study. Ann Intensive Care.

[24] Ikeda S, Misumi T, Izumi S, Sakamoto K, Nishimura N, Ro S (2021). Corticosteroids for hospitalized patients with mild to critically-ill COVID-19: a multicenter, retrospective, propensity score-matched study. Sci Rep.

[25] Gragueb-Chatti I, Lopez A, Hamidi D, Guervilly C, Loundou A, Daviet F (2021). Impact of dexamethasone on the incidence of ventilator-associated pneumonia and blood stream infections in COVID-19 patients requiring invasive mechanical ventilation: a multicenter retrospective study. Ann Intensive Care.

[26] Reyes LF, Rodriguez A, Bastidas A, Parra-Tanoux D, Fuentes YV, García-Gallo E (2022). Dexamethasone as risk-factor for ICU-acquired respiratory tract infections in severe COVID-19. J Crit Care.

[27] Dupuis C, de Montmollin E, Buetti N, Goldgran-Toledano D, Reignier J, Schwebel C (2021). Impact of early corticosteroids on 60-day mortality in critically ill patients with COVID-19: A multicenter cohort study of the OUTCOMEREA network. PLoS ONE.

[28] Rodríguez A, Ruiz-Botella M, Martín-Loeches I, Jimenez Herrera M, Solé-Violan J, Gómez J (2021). Deploying unsupervised clustering analysis to derive clinical phenotypes and risk factors associated with mortality risk in 2022 critically ill patients with COVID-19 in Spain. Crit Care Lond Engl.

[29] Lascarrou J-B, Gaultier A, Soumagne T, Serck N, Sauneuf B, Piagnerelli M (2021). Identifying clinical phenotypes in moderate to severe acute respiratory distress syndrome related to COVID-19: the COVADIS study. Front Med.

[30] Bos LDJ, Sjoding M, Sinha P, Bhavani SV, Lyons PG, Bewley AF (2021). Longitudinal respiratory subphenotypes in patients with COVID-19-related acute respiratory distress syndrome: results from three observational cohorts. Lancet Respir Med.

[31] Lascarrou J-B (2021). COVID-19-related ARDS: one disease, two trajectories, and several unanswered questions. Lancet Respir Med.

[32] Mongardon N, Piagnerelli M, Grimaldi D, Perrot B, Lascarrou JB (2020). Impact of late administration of corticosteroids in COVID-19 ARDS. Intensive Care Med.

[33] Villar J, Confalonieri M, Pastores SM, Meduri GU (2020). Rationale for prolonged corticosteroid treatment in the acute respiratory distress syndrome caused by coronavirus disease 2019. Crit Care Explor.

[34] Surveillance of healthcare-associated infections in intensive care units—Publications Office of the EU [cited 2022 Jan 26]. https://op.europa.eu/en/publication-detail/-/publication/803d18a8-82f7-11e7-b5c6-01aa75ed71a1/language-en.

[35] Fernando SM, Tran A, Cheng W, Klompas M, Kyeremanteng K, Mehta S (2020). Diagnosis of ventilator-associated pneumonia in critically ill adult patients—a systematic review and meta-analysis. Intensive Care Med.

