# Supplementary material for: Early steroids and ventilator-associated pneumonia in COVID-19-related ARDS
Source: Crit Care. 2022 Aug 2;26:233. doi: 10.1186/s13054-022-04097-8 (PMC9344449; doi:10.1186/s13054-022-04097-8)
Supplement: Supplementary file 1 — Additional file 1: Supplementary methods, eFigure 1, eTable 1 and eFigure 2. [file 13054_2022_4097_MOESM1_ESM.docx]

**SUPPLEMENTARY DATA**

**Methods**

***Design and population***

We retrospectively collected data for patients admitted to any of 15 ICUs in Western France, from February 1, 2020, to December 31, 2020. The data for each patient were entered into an electronic case report form (Castor EDC, Amsterdam, The Netherlands) by the investigators in each participating center.

Patients older than 18 years old were eligible for inclusion if they required invasive mechanical ventilation (MV) for longer than 48 h to treat acute respiratory distress syndrome and had a positive reverse transcriptase-polymerase chain reaction test for SARS-CoV-2 in one or more upper and/or lower respiratory tract samples. We did not include pregnant women or patients under guardianship.

The main study outcome was the cumulative incidence of ventilator-associated pneumonia (VAP), which was compared in the group given early corticosteroid treatment (cases) and in the group given no or only delayed corticosteroid treatment (controls). Secondary outcomes were day-90 mortality, MV duration, other organ dysfunctions, and VAP characteristics.

***Data collection***

The following data were collected for each patient: age, sex, and body mass index; Charlson’s Comorbidity Index [1]; date of SARS-CoV-2 symptom onset; ventilatory support on the first ICU day; date of MV initiation; whether Berlin criteria for ARDS were met [2]; presence of coinfection; need for veno-venous extracorporeal membrane oxygenation and/or vasopressor therapy; use of antiviral agents, immunomodulatory drugs, and/or initial antibiotics; and VAP characteristics (time of onset, sampling method, causative microorganisms, and complications) with follow-up until day 90 or death. The type, dose, and duration of corticosteroid treatment were also recorded. Illness severity was assessed by determining the Simplified Acute Physiology Score (SAPS) II score [3] and organ failure by determining the Sequential Organ Failure Assessment (SOFA) [18], 24 h after ICU admission.

***Definitions***

We defined early corticosteroid treatment as the administration of systemic corticosteroids before or within 24 h after ICU admission.

Patients were classified as immunocompromised if they had any of the following: chemotherapy within the past 3 months to treat a solid malignancy, progressive metastatic disease, hematologic malignancy, solid-organ transplantation, HIV infection with or without AIDS, corticosteroid treatment for longer than 3 months in a dosage of at least 1 mg/kg/day prednisolone-equivalent for longer than 7 days, and treatment with another immunosuppressive drug [4].

The risk factors for multidrug-resistant (MDR) pathogens were defined as antibiotic therapy, hospitalization for longer than 48 h within the last 3 months, and known MDR carriage [5].

***Management of ventilator-associated pneumonia (VAP)***

The diagnosis of VAP was established by the team in charge of the patient. VAP was defined as hospital-acquired pneumonia diagnosed after at least 48 h of MV. In extubated patients, hospital-acquired pneumonia was categorized as VAP if onset occurred within 48 h after extubation. The diagnostic criteria were those recommended by the European Centre for Disease Prevention and Control (ECDC) [6], i.e., two consecutive chest radiographs or computed tomography scans showing new or progressive lung infiltrates combined with one or more systemic signs (body temperature >38.3 °C not due to another cause and/or leukocyte count <4000/mm^3^ or >12 000/mm^3^), one or more respiratory signs (new onset of purulent sputum or change in character of sputum [color, odor, quantity, consistency] and/or worsening gas exchange [blood oxygen desaturation or increased oxygen requirements or increased ventilation requirements]); and one or more microbiological signs (positive quantitative culture from a minimally contaminated lower respiratory tract specimen [PN 1 type in the ECDC classification] obtained using a plugged telescopic catheter, with a threshold of 10^3^ colony-forming units/mL; or from a bronchoalveolar-lavage specimen, with a threshold of 10^4^ colony-forming units/mL; or from a possibly contaminated lower respiratory tract specimen [PN 2 type in the ECDC classification] consisting in a blind endotracheal aspirate, with a threshold of 10^6^ colony-forming units/mL; or positive culture of a pleural fluid specimen (PN 3 type in the ECDC classification).

Local protocols to minimize the incidence of VAP included oral instead of nasal intubation when possible, head-of-bed elevation to 30°–45°, periodic suctioning-system drainage with discarding of the condensate in the tubing, use of a new ventilator circuit for each patient, circuit change only if soiling or damage occurs (not routinely), heat-moisture exchanger replacement every 5–7 days or in the event of soiling or malfunction, and daily oral hygiene [22].

**eFigure 1: Microorganisms recovered in patients with ventilator-associated pneumonia who were vs. were not given early corticosteroid treatment**

**eTable 1: Bloodstream infection characteristics in patients who were vs. were not given early corticosteroid treatment**

|  | Total  (N=670) | Early CS^a^  (N=369) | No early CS^a^  (N=301) | *P* value |
| --- | --- | --- | --- | --- |
| Bloodstream infection, n (%) | 113 (16.0) | 73 (18.7) | 40 (12.7) | 0.0303 |
| Pulmonary bacteremia source, n (%) | 35 (5.0) | 23 (6.0) | 12 (3.8) | 0.1891 |
| **Bacterial antibiotic resistance profile** | | | | |
| Normal, n (%) | 68 (61.3) | 45 (62.5) | 23 (59.0) | 0.0751 |
| MDR, n (%) | 40 (36.0) | 27 (37.5) | 13 (33.3) |  |
| XDR, n (%) | 3 (2.7) | 0 (0.0) | 3 (7.69) |  |
| PDR, n (%) | 0 (0.0) | 0 (0.0) | 0 (0.0) |  |

CS: corticosteroid therapy; MDR: multidrug resistant; XDR: extensively drug resistant; PDR: pandrug resistant

^a^Early CS was defined as the administration of systemic corticosteroid therapy before ICU admission or within 24 hours after ICU admission.

**eFigure 2. Survival probability in patients who were vs. were not given early corticosteroid treatment**

**References**

1. Brusselaers N, Lagergren J. The Charlson Comorbidity Index in registry-based research. Methods Inf Med. 2017;56:401–6.

2. ARDS Definition Task Force, Ranieri VM, Rubenfeld GD, Thompson BT, Ferguson ND, Caldwell E, et al. Acute respiratory distress syndrome: the Berlin Definition. JAMA. 2012;307:2526–33.

3. Le Gall JR, Lemeshow S, Saulnier F. A new Simplified Acute Physiology Score (SAPS II) based on a European/North American multicenter study. JAMA. 1993;270:2957–2963.

4. Vincent JL, Moreno R, Takala J, Willatts S, De Mendonça A, Bruining H, et al. The SOFA (Sepsis-related Organ Failure Assessment) score to describe organ dysfunction/failure. On behalf of the Working Group on Sepsis-Related Problems of the European Society of Intensive Care Medicine. Intensive Care Med. 1996;22:707–10.

5. Azoulay E, Lemiale V, Mokart D, Nseir S, Argaud L, Pène F, et al. Effect of high-flow nasal oxygen vs standard oxygen on 28-day mortality in immunocompromised patients with acute respiratory failure: The HIGH randomized clinical trial. JAMA. 2018;320:2099–2107.

6. Controversies in guidelines for the control of multidrug-resistant Gram-negative bacteria in EU countries - ScienceDirect [Internet]. [cited 2022 Jan 27]. Available from: https://www.sciencedirect.com/science/article/pii/S1198743X15008782?via%3Dihub

7. Surveillance of healthcare-associated infections in intensive care units - Publications Office of the EU [Internet]. [cited 2022 Jan 26]. Available from: https://op.europa.eu/en/publication-detail/-/publication/803d18a8-82f7-11e7-b5c6-01aa75ed71a1/language-en
